# A conditionally replicative adenovirus vector containing the *synNotch* receptor gene for the treatment of muscle-invasive bladder cancer

**DOI:** 10.1038/s41417-025-00879-8

**Published:** 2025-02-26

**Authors:** Ruhan A, Hideto Ueki, Shunya Nishioka, Rion Yamazaki, Marina Maekawa, Koichi Kitagawa, Hideaki Miyake, Toshiro Shirakawa

**Affiliations:** 1 Department of Advanced Medical Science, Kobe University Graduate School of Science, Technology and Innovation, Kobe, Japan; 2 Division of Urology, Kobe University Graduate School of Medicine, Kobe, Japan

**Keywords:** Bladder cancer, Cancer stem cells, Drug development

## Abstract

Muscle-invasive bladder cancer (MIBC), a highly heterogeneous disease, shows genomic instability and a high mutation rate, making it difficult to treat. Recent studies revealed that cancer stem cells (CSCs) play a critical role in MIBC frequent recurrence and high morbidity. Previous research has shown that Cyclooxygenases-2 (COX-2) is particularly highly expressed in bladder cancer cells. In recent years, the development of oncolytic adenoviruses and their use in clinical trials have gained increased attention. In this study, we composed a conditionally replicative adenovirus vector (CRAd-synNotch) that carries the COX-2 promotor driving adenoviral *E1* gene, the *synNotch* receptor therapeutic gene, and the *Ad5/35 fiber* gene. Activation of the COX-2 promoter gene causes replication only within COX-2 expressing cancer cells, thereby leading to tumor oncolysis. Also, CD44 and HIF signals contribute to cancer stemness and maintaining CSCs in bladder cancer, and the transduced synNotch receptor inhibits both CD44 and HIF signals simultaneously. We performed an in vivo study using a mouse xenograft model of T24 human MIBC cells and confirmed the significant antitumor activity of CRAd-synNotch. Our findings in this study warrant the further development of CRAd-synNotch for treating patients with MIBC.

## Introduction

Bladder cancer (BC) is the tenth most commonly diagnosed cancer in the world and is often characterized by its tendency to recur. The annual incidence worldwide was ~570,000 cases in 2020, with around 210,000 deaths reported [1]. The depth of cancer infiltration determines treatment for bladder cancer. Cancer confined to the epithelium is diagnosed as non-muscle invasive bladder cancer (NMIBC). Approximately 70% of patients fall into this category. When cancer has invaded the muscle layer, it is diagnosed as muscle invasive bladder cancer (MIBC), and ~25% of patients fall into this category and 5% have distant metastases [1, 2]. The standard of care for MIBC is radical cystectomy, followed by urinary diversion surgery. However, due to the low 5 year survival rate of ~50% with cystectomy alone, neoadjuvant chemotherapy with cisplatin may be administered prior to surgery to improve treatment outcomes. This approach is curative in intent. Nevertheless, it is estimated that around 50% of MIBC patients do not undergo radical cystectomy [3, 4]. The 5 year overall survival rate for patients with metastatic disease is <10% [5]. Despite the introduction of novel treatments for MIBC in the past few years, such as therapies targeting the fibroblast growth factor receptor (FGFR), anti–PD-(L)1 antibodies, and antibody-drug conjugates, the primary approach to treatment has remained radical cystectomy. MIBC is identified as the most challenging molecular subtype of bladder cancer to treat. Establishing an effective therapeutic modality for MIBC is an urgent unmet medical need.

Cancer stem cells (CSCs) play an essential role in the acquisition of chemoresistance [6, 7], and thus the development of novel MIBC therapies targeting bladder cancer stem cells (BCSCs) is attracting significant attention [8]. CD44 is a receptor on the cell surface for binding extracellular matrix proteins, such as hyaluronic acid (HA), and is recognized as a marker for cancer stem cells in bladder cancer [9]. Recent studies have indicated the close association of CD44 with the metastatic ability and stemness of bladder cancer cells [10]. It is markedly linked to a more advanced clinical tumor stage, involvement of lymph nodes, locoregional relapse, and diminished survival rates in patients with MIBC undergoing curative therapy [11].

Almost all solid tumors have a hypoxic environment due to the imbalance between cell proliferation and angiogenesis. Several studies reported that hypoxia-inducible factor-1 (HIF-1) is the central mediator of adaptive responses to hypoxia in bladder cancer [12, 13]. Under normoxia conditions, HIF-1α turns over very quickly, being hydroxylated by Prolyl Hydroxylase Domain protein (PHD) and then bound by the Von Hippel-Lindau (VHL) protein for degradation in the 26S proteasome. In hypoxia conditions, the activity of PHD is reduced, preventing the degradation of HIF-1α. The accumulated HIF-1α then translocates to the nucleus, forms a heterodimer with HIF-1β and binds to hypoxia-responsive elements of genes to activate the transcription of various hypoxia target genes [14], promoting tumorigenesis and CSC maintenance [15]. One of the HIFs, HIF-3α, exists as multiple splice variants, and some variants, including HIF-3α4, inhibit binding to DNA through interaction with HIF-1α and thus suppress the function of HIF-1α [16, 17].

**Fig. 1:**
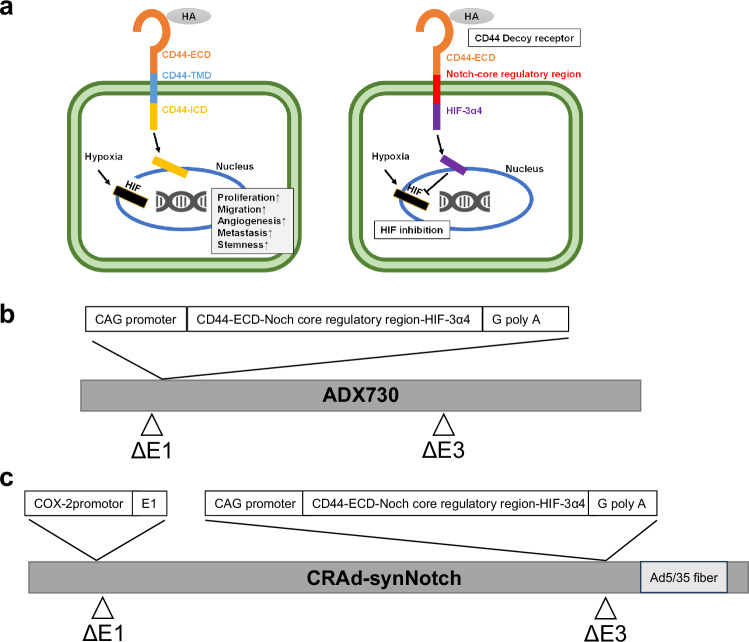
Construction of ADX730 and CRAd-synNotch. Construction of recombinant replication-deficient adenoviral vector. A synthetic fusion gene encoding a synNotch receptor (**a**) was introduced into the ΔE1 region of the adenovirus type 5 vector to construct a recombinant replication-deficient adenoviral vector, ADX730 (**b**). Construction of conditionally replicative adenovirus vector. (CRAd-synNotch) carries the COX-2 promotor gene upstream of the E1 region of the adenovirus vector genome, the synNotch receptor gene in the E3 region, and the Ad5/35 fiber gene downstream of the E3 region (**c**).

In recent years, a growing focus has been on developing oncolytic viruses and their application in clinical studies. Adenoviruses stand out as some of the most extensively researched viruses in the realm of oncolytic virotherapy, with numerous molecular biological breakthroughs originating from studies of adenoviruses [18, 19]. Recent studies revealed that CSCs play a critical role in MIBC frequent recurrence, metastasis and high morbidity [20, 21]. Thus, a novel therapy targeting CSCs has great potential to suppress the acquisition of MIBC recurrence and morbidity [22]. It is well known that both CD44 and HIF contribute to cancer stemness in the tumor microenvironment [23, 24]. To target both CD44 and HIF simultaneously, we constructed a recombinant replication-deficient adenovirus vector (ADX730) containing a gene encoding a synthetic Notch (synNotch) receptor (CD44-N-HIF3α4) composed of the extracellular domain of CD44 (CD44-ECD) and HIF-3α4 connected by the Notch core regulatory region in previous research (Fig. 1a and b). The mechanism of the synNotch receptor was explained previously [25]. ADX730 can induce overexpression of the synNotch receptor of CD44-N-HIF3α4 in cancer cells and showed high antitumor efficacy in breast cancer [25].

In this study, we composed a conditionally replicative adenovirus vector (CRAd-synNotch) that carries the COX-2 promotor driving adenoviral *E1* gene, the *synNotch* receptor therapeutic gene, and the *Ad5/35 fiber* gene (Fig. 1c). The COX-2 promotor gene is a promoter that becomes transcriptionally active only in Cyclooxygenases-2 (COX-2) expressing cells. Previous research has shown that COX-2 is particularly highly expressed in bladder cancer cells, suggesting that the COX-2 promotor gene may exhibit cancer cell-selective transcriptional activity [26]. CRAd-synNotch introduces the COX-2 promotor gene upstream of the E1 region, which is responsible for amplifying the adenovirus vector, thereby causing replication only within COX-2 expressing cancer cells and leading to tumor oncolysis. Type 5 adenoviruses enter cells through the Coxsackievirus and adenovirus receptor (CAR) on the cell surface, but the level of CAR expression is inversely correlated with the malignancy of the tumor, potentially reducing injection efficiency based on the malignancy of the cancer [27]. On the other hand, CD46, a cell surface protein, functions as a complement inhibitor, and its expression level is known to correlate with the malignancy of bladder cancer [28]. By using the Ad5/35 fiber on the surface of the adenovirus vector, it is possible to enter cells from both CAR and CD46 [28, 29]. Thus, CRAd-synNotch is expected to not only exhibit antitumor effects through the synNotch gene but also enable tumor dissolution due to the activity of the COX-2 promotor gene, and these effects are anticipated to occur regardless of the malignancy level of the cancer.

In the present study, we explored the feasibility of using CRAd-synNotch to treat MIBC by in vitro and in vivo experiments using T24 human MIBC cells. We compared its in vivo antitumor activities with control conditionally replicative adenovirus, CRAd-GFP and ADX730. CRAd-GFP, a conditionally replicative adenovirus vector, carries the COX-2 promotor gene upstream of the E1 region of the adenovirus vector genome, the AcGFP1 DNA in the E3 region, and the Ad5/35 fiber gene downstream of the E3 region. Our results confirmed that CRAd-synNotch showed the strongest anti-tumor activity in the T24 xenograft model in vivo compared to CRAd-GFP and ADX730.

## Materials and methods

### Cell lines

T24, a human MIBC cell line, was purchased from the American Type Culture Collection (ATCC, Manassas, VA) and cultured in Eagle’s minimal essential medium (E-MEM, FUJIFILM Wako Pure Chemical Corporation, Osaka, Japan) supplemented with 10% fetal bovine serum (FBS; Sigma Aldrich, St. Louis, MO), 100 U/mL penicillin, and 100 μg/mL streptomycin (P/S; Nacalai Tesque, Kyoto, Japan). SV-HUC-1, a human uroepithelium cell line was purchased from ATCC and cultured in Dulbecco’s modified Eagle’s medium (DMEM, FUJIFILM Wako Pure Chemical Corporation) supplemented with 10% FBS, 100 U/mL penicillin, and 100 μg/mL streptomycin. BT474, a COX-2 negative human breast cancer cell line [30], was purchased from ATCC and cultured in DMEM supplemented with 10% FBS, 100 U/mL penicillin, and 100 μg/mL streptomycin. The human bladder cancer cell lines KK47 and 5637 were purchased from ATCC and cultured in Roswell Park Memorial Institute 1640 Medium (RPMI 1640, FUJIFILM Wako Pure Chemical Corporation) supplemented with 10% FBS, 100 U/mL penicillin, and 100 μg/mL streptomycin. All cells were cultured at 37°C in a humidified atmosphere of 5% CO_2_. To generate hypoxic (2% O_2_) culture conditions, we used Multi-gas-incubators (PHC Holdings Corporation, Tokyo, Japan).

### Construction of CRAd-synNotch and CRAd-GFP

The vector DNA used in this study included plasmids pAd1127-04, pAd1128, pAd1129-06, and pAd1130-04 provided with the AdenoQuick 2.0 Kit (O.D.260Inc, Boise, ID). Additionally, the Cosmid Construction Kit-2 (O.D.260Inc) was used to create cosmid vectors. The *synNotch* receptor gene, recombinant adenovirus vector ADX730, and Ad-LacZ were constructed as previously described [25]. The insert PCR was performed using KOD-Plus-Neo (TOYOBO, Osaka, Japan) with pDrive-hCOX2 (InvivoGen, San Diego, CA), amplifying only the Cox-2 promoter gene. The COX-2 DNA was digested using SalI (TaKaRa Bio Inc., Shiga, Japan) and EcoRI (TaKaRa) restriction enzymes, followed by agarose gel electrophoresis and purification, and insertion into pAd1127-04. フォームの始まりThe construction of the *synNotch* receptor gene was outsourced to Gene Universal (Newark, DE). The product obtained from the restriction enzyme treatment with EcoRI (TaKaRa), electrophoresis, and purification was inserted into pAd1129-06. The plasmids pAd1127-04, pAd1129-06, pAd1128 (O.D.260Inc), and pAd1130-04 (O.D.260Inc), each containing the insert DNA, were subjected to restriction enzyme treatment using SfiI (New England Biolabs, Ipswich, MA). After agarose gel electrophoresis, each DNA fragment was purified, and after the ligation reaction a cosmid vector was constructed. The constructed cosmid vector was used as vector DNA for constructing CRAd-synNotch. CRAd-synNotch was constructed by transfecting this vector DNA to HEK293 cells. The abovementioned procedures were conducted per the manual of the AdenoQuick 2.0 Kit (O.D.260Inc) and Cosmid Construction Kit-2. CRAd-GFP, a conditionally-replicative adenovirus vector serving as a negative control for CRAd-synNotch, was constructed by substituting the insert DNA of CRAd-synNotch with AcGFP1 DNA to generate an adenovirus vector. The virus was amplified in HEK293 cells and purified using CsCl_2_ step gradient ultracentrifugation followed by CsCl_2_ linear gradient ultracentrifugation. The purified viruses were subjected to buffer exchange using Amicon Ultra-15 (Merck Millipore, Burlington, MA) into a dispersion medium at pH 8.0 containing Tris (FUJIFILM Wako Pure Chemical Corporation), sucrose (FUJIFILM Wako Pure Chemical Corporation), NaCl (Nacalai Tesque), MgCl2 (FUJIFILM Wako Pure Chemical Corporation), Tween 80 (FUJIFILM Wako Pure Chemical Corporation), EDTA (Nacalai Tesque), and ethanol (FUJIFILM Wako Pure Chemical Corporation) by standard procedures. The solutions were aliquoted appropriately and stored at -80°C [29]. A standard plaque-forming assay determined viral particle and biological titers [31, 32]. This study was approved by the Committee for Safe Handling of Living Modified Organisms of Kobe University and carried out according to the committee guidelines.

### Flow cytometry

The expressions of Coxsackievirus and adenovirus receptor (CAR), CD46, and CD44 on the surface of BT474 and T24 cells were assessed by flow cytometry. Briefly, the cells (1 × 10^6^ cells/well) were seeded in 6-well flat bottom culture plates (Corning, Corning, NY), and then washed with phosphate buffer solution (PBS) before retrieval. Blocking One Histo (Nacalai Tesque, Inc., Kyoto, Japan) was used to conduct 10 min blocking at room temperature. Cells were re-washed with PBS after blocking. The PE-Anti-CAR (Merck Millipore, Burlington, MA), FITC ANTI-HUMAN CD46 (BioLegend), and APC ANTI-MOUSE/HUMAN CD44 (BioLegend) were added for a 30 min reaction on ice. Cells were re-washed with PBS after the reaction and 100 fold-diluted BD Pharmingen™ 7-AAD (BD Biosciences, San Diego, CA) was added for a 5 min reaction on ice under light-resistant conditions. Cells were re-washed with PBS after this reaction. Fusion gene expression was determined by Guava® easyCyte™ (Merck Millipore, Burlington, MA), and data were analyzed with InCyte software. All these results were expressed as the mean ± SE for three separate experiments.

### Adenovirus vector replication experiment

ADX730, CRAd-GFP, CRAd-synNotch, and a non-genetically modified type 5 wild-type adenovirus (wtAd) demonstrating Cox-2-independent proliferation were used in the following experiments. wtAd, ADX730, CRAd-GFP, and CRAd-synNotch were used to infect COX-2-positive cell lines KK47, 5637, T24 and COX-2-negative cell lines BT474, and PCR evaluated the viral replication capacity driven by the COX-2 promoter and E1 promoter in vitro. KK47, 5637, T24 and BT474 cell lines were seeded at a density of 5 × 10^5^ cells/well in a 6-well flat-bottom cell culture plate (CORNING) and cultured overnight at 37°C with 5% CO_2_. After culturing, the cells were infected with 50 MOIs of wtAd, ADX730, CRAd-GFP, and CRAd-synNotch for 1 h at 37°C with 5% CO_2_. After infection, cells were cultured for another 24 h at 37°C with 5% CO_2_. After culturing, the cells were harvested and DNA extraction was performed using NucleoSpin® Tissue (MACHEREY-NAGEL, Duren, Germany). The extracted DNA was quantified and analyzed by PCR using TB GreenTM Premix Ex TaqTM II (TaKaRa). All these results were expressed as the mean ± SE for three separate experiments.

### Western blotting

T24 cells were plated in 6-well plates at 5 × 10^5^ cells/well density and incubated at 37°C and 5% CO_2_ for 24 h. Then the cells were infected with ADX730 100 MOIs and incubated for an additional 48 h and 72 h. The cells infected with 50 MOIs of CRAd-synNotch were cultured for an additional 24 h and 72 h. These cells were harvested and washed by PBS, then lysed in 8 M urea buffer containing 0.1% dithiothreitol, and protein concentration was determined. Equal amounts of each sample were added into the sample buffer (Nacalai Tesque) and heated at 95°C for 5 min. The samples were separated by SDS-PAGE and transferred to a polyvinylidene difluoride membrane. After blocking with Blocking One (Nacalai Tesque) 1 h at room temperature (RT), followed by washing, the membranes were incubated overnight at RT with anti-CD44 (E7K2Y) monoclonal antibody (Catalog#: 37259S, Cell Signaling Technology, Danvers, MA), 1:1000, HIF3α4 polyclonal antibody (Hokudo Co., Ltd., Hokkaido, Japan) or anti-beta-actin antibody (Catalog#: sc-47778, Santa Cruz Biotechnology, Dallas, TX), 1:1000. The CD44 antibody was diluted with Can Get Signal Immunoreaction Enhancer Solution (TOYOBO, Osaka, Japan), and HIF3α4 and beta-actin antibodies were diluted with PBS-Tween 20%. After another washing, membranes were incubated for 1 h at RT with HRP conjugated goat anti-mouse IgG or anti-rabbit IgG 1:1000. Antibody binding to proteins was detected by enhanced chemiluminescence.

### Real-time reverse transcriptase-polymerase chain reaction (RT-PCR)

Real-time RT-PCR examined the expression of HIF-3α4 mRNA levels in T24 cells and gene expressions of hyaluronan synthase *(HAS) 1*, *HAS2, HAS3* and *COX-2* mRNAs in KK47, 5637, T24 and SV-HUC-1 cell lines. Gene transduction with recombinant adenoviral vectors, including synNotch receptor, and resulting antitumor effects were verified by real-time RT-PCR. Briefly, the cells (1 × 10^6^ cells/well) were seeded in 6-well flat-bottomed culture plates (Corning Inc.) and cultured overnight at 37°C and 5% CO_2_ under conditions of normoxia (21%) or hypoxia (2%). Multi-gas-incubators (PHC Holdings Corporation) were used to generate hypoxia (2%). The cells were infected with Ad-LacZ and ADX730 at a multiplicity of infection (MOI) of 100/cell and infected with CRAd-GFP and CRAd-synNotch respectively at 50 MOIs /cell. Cells were incubated for another 48 h and 72 h then retrieved to extract total RNA using NucleoSpin® RNA (TaKaRa Bio). cDNA was synthesized from extracted RNA using the PrimeScript™ RT Regent Kit with gDNA Eraser (TaKaRa Bio Inc.). The primers (Table 1), TB Green™ Prime Ex Ta™ (TaKaRa Bio Inc.), and Thermal Cycler Dice® Real Time System (TaKaRa Bio Inc.) were used to conduct real-time RT-PCR before analyses according to the ΔΔCt method. All these results were expressed as the mean ± SE for three separate experiments.

**Table 1:** Primer sequences for Real-time RT-PCR.

*Gene*	Sequences
*COX-2*	Forward: 5’-GATACTCAGGCAGAGATGATCTACCC-3’
	Reverse: 5’-AGACCAGGCACCAGACCAAAGA-3’
*CD44*	Forward: 5’-TGCCGCTTTGCAGGTGTATT-3'
	Reverse: 5’-CCGATGCTCAGAGCTTTCTCC-3'
*HAS1*	Forward: 5’-GGAATAACCTCTTGCAGCAGTTTC-3’
	Reverse: 5’-GCCGGTCATCCCCAAAAG-3’
*HAS2*	Forward: 5’-TCGCAACACGTAACGCAAT-3’
	Reverse: 5’-ACTTCTCTTTTTCCACCCCATTT-3’
*HAS3*	Forward: 5’-AACAAGTACGACTCATGGATTTCCT-3’
	Reverse: 5’-GCCCGCTCCACGTTGA-3’
*HIF-3α4*	Forward: 5’-GGGAGACATGGCTTACCTGT-3'
	Reverse: 5’-GCGTACTCTTCATGCGCAAG-3'
*SOX-2*	Forward: 5’-GACAGTTACGCGCACATGAA-3'
	Reverse: 5’-TAGGTCTG CGAGCTGGTCAT-3'
*Cortactin*	Forward: 5’-AAAGCTTCAGCAGGCCAC-3'
	Reverse: 5’-TTTGGTCCTGTTTCAAGTTCC-3'
*OCT4*	Forward: 5’-GGTATTCAGCCAAACGACCA-3'
	Reverse: 5’-CACACTCGGACCACATCCTT-3'
*Nanog*	Forward: 5’-GTGATTTGTGGGCCTGAAGA-3
	Reverse: 5’-ACACAGCTGGGTGGAAGAGA-3'
c-Myc	Forward: 5’-TGGTCTTCCCCTACCCTCTCAAC-3
	Reverse: 5’-GATCCAGACTCTGACCTTTTGCC-3'
Cyclin-d1	Forward: 5’-CTGTGCTGCGAAGTGGAAACCAT-3
	Reverse: 5’-TTCATGGCCAGCGGGAAGACCTC-3'
*VEGF*	Forward: 5’-TACCTCCACCATGCCAAGTG-3
	Reverse: 5’-ATGATTCTGCCCTCCTCCTTC-3'
*PHD3*	Forward: 5’-TCCTGCGGATATTTCCAGAGG-3
	Reverse: 5’- GGTTCCTACGATCTGACCAGAA-3'
*GLUT1*	Forward: 5’-CCAGGGTAGCTGCTGGAGC-3
	Reverse: 5’- TGGCATGGCGGGTTGT-3'
*Bcl-xL*	Forward: 5’-CCCAGA AAGGATACAGCTGG-3'
	Reverse: 5’-GCGATCCGACTCACCAATAC-3'
*CyclinG2*	Forward: 5’-GCTGAAAGCTTGCAACTGCCGAC-3'
	Reverse: 5’-GGTATCGTTGGCAGCTCAGGAAC-3'
*TBP*	Forward: 5’-GCCAGCTTCGGAGAGTTCTGGGATT-3’
	Reverse: 5’-CGGGCACGAAGTCAATGGTCTTTA-3’

### Cell proliferation assay

BT474 and T24 cells were seeded at a density of 5.0 × 10³ cells/well in a 96-well plate (Corning Inc.) and cultured for 24 h. Then the cells were treated with 50 MOIs of ADX730, CRAd-GFP, and CRAd-synNotch and incubated at 37°C for 72 h at 2% or 21% oxygen concentration. Then colorimetric reagents Cell Titer 96 Aqueous One Solution Cell Proliferation Assay (Promega, Madison, WI) were added and the absorbance was measured at a wavelength of 492 nm using a microplate photometer (Thermo Fisher Scientific). All these results were expressed as the mean ± SE for three separate experiments.

### Animal studies

In an in vivo study, the antitumor effect of CRAd-synNotch was confirmed compared to the control adenoviral vector CRAd-GFP, ADX730, and PBS group. Briefly, the mixture of T24 cells (1 × 10^6^ cells/70 μL) and 70 μL of Matrigel® Matrix Basement Membrane HC (Corning, Inc.) were subcutaneously inoculated into the right lumbar region of 20 female BALB/c-nu /nu mice aged 6 weeks (CLEA Japan, Inc., Tokyo, Japan). Tumor implantation was verified on day 13 after xenografting, and the 20 mice were randomly allocated to 4 groups (with 5 mice used per group) in a blinded manner. The treatment groups were established for a total of 6 intratumoral injections of adenoviral vectors and control on alternate days (the start day of injection 0, 4, 8, 12, 16 and 20) as PBS control (50 μL), ADX730, CRAd-GFP and CRAd-synNotch (1 × 10^9^ IFU/50 µL each). Tumor diameters were measured twice weekly from the start day of injection. The major (L) and minor (W) axes of the tumor were measured to calculate tumor volume according to the formula (W^2^ × L)/2. After treatment completion, survival was also observed.

### Immunohistochemical staining

Tumor infected tissue with PBS, CRAd-GFP, ADX730 and CRAd-synNotch was resected and fixed with paraformaldehyde. Paraffin-embedded T24 tumor tissue sections were deparaffinized and rehydrated. Antigen retrieval was performed in Bond epitope retrieval buffer (pH6.0; Leica Microsystems, Wetzlar, Germany) at 98°C for 20 min according to the manufacturer’s standard protocol. Immunohistochemical staining was performed in an automatic tissue processor (Leica Microsystems Bond). Briefly, tissue sections were incubated at RT for 15 min with an anti-CD44 antibody (1:600, Catalog#: 37259S, Cell Signaling Technology). After washing, sections were incubated with horseradish peroxidase-conjugated secondary antibodies. After washing, sections were incubated with 3,3’-diaminobenzidine (Muto Pure Chemicals Co., Ltd., Tokyo, Japan) and counterstained with hematoxylin. The resulting tissue slides were observed under a BZ-X710 microscope (Keyence, Osaka, Japan).

### Statistical analysis

Comparisons between two groups were performed using a two-tailed student’s *t*-test, and comparisons between multiple groups were performed using one-way ANOVA followed by the Tukey–Kramer method. Differences among experimental groups were considered significant when *p* < 0.05. Data are presented as mean ± standard error (SE).　The power analysis approach calculated the sample size of the animal study. Overall survival rates were estimated using the Kaplan–Meier method, with statistical comparisons conducted using the Log-rank test. Criteria for excluding samples from analysis were established in advance. For example, animals that died during the experiment or cell samples in which appropriate gene expression could not be confirmed were excluded.

### Ethical statement

All experiments pursued the standards of the National Institutes of Health Guide for the Care and Use of Laboratory Animals (NIH Publications No. 8023, revised 1978). The experimental protocol was approved by the Institutional Animal Experimentation Regulations. The approval number is P201108, and the approval date is December. 23, 2020. The study also follows ARRIVE guidelines and complies with the National Research Council’s guide for the care and use of laboratory animals

## Results

### T24 cells express CAR, CD46 and CD44

**Fig. 2:**
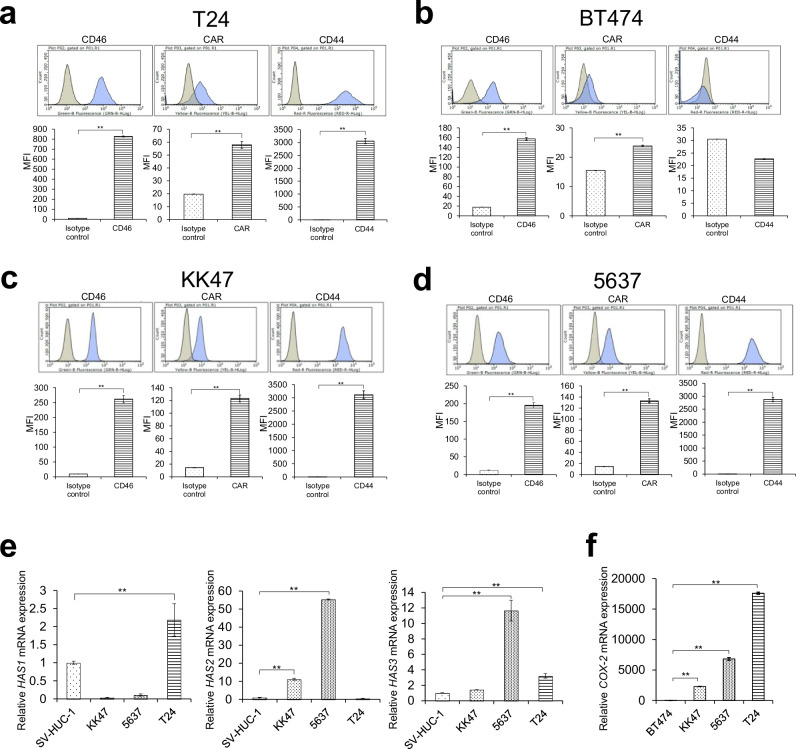
Expression of CD46, CAR, CD44 and induction of hyaluronan synthases (HAS1, HAS2, and HAS3) and COX-2 mRNA in KK47, 5637 and T24 cells. **a** The expression of CD46, CAR, and CD44 on the surface of T24 cells was evaluated by flow cytometry. **b** The expression of CD46, CAR, and CD44 on the surface of BT474 cells was evaluated by flow cytometry. **c** The expression of CD46, CAR, and CD44 on the surface of KK47 cells was evaluated by flow cytometry. **d** The expression of CD46, CAR, and CD44 on the surface of 5637 cells was evaluated by flow cytometry. **e** The mRNA expressions of HAS1, HAS2 and HAS3 in KK47, 5637 and T24 cells were measured by real-time RT-PCR. **f** The mRNA expressions of COX-2 in KK47, 5637 and T24 cells were measured by real-time RT-PCR. All mRNA levels were standardized by the expression levels of the control gene TATA-binding protein (TBP) and analyzed using the ΔΔCt method. All these results were expressed as the mean ± SE for three separate experiments (*p < 0.05, **p < 0.01).

Before investigating the antitumor activity of CRAd-synNotch and ADX730 in T24 (MIBC) and BT474 (a COX-2 negative human breast cancer cell line) cells, we confirmed the expressions of CAR, CD46 and CD44 expressions on the cell surface. The expression of CAR and CD46 on the cell surface is strongly correlated with the infectivity of adenovirus type 5 [29, 33], and thus confirmation of cell-surface expression of CAR and CD46 proteins is important for the further evaluation of CRAd-synNotch and ADX730. The significantly higher expressions of CAR and CD46 in KK47, 5637, T24 and BT474 [30] compared to each isotype control were confirmed by flow cytometry (*p* < 0.01; Fig. 2a–d). Furthermore, CD44 was highly expressed in KK47, 5637, and T24, but not in BT474 (*p* < 0.01; Fig. 2a–d). These results indicate that the KK47, 5637, T24 and BT474 cell lines are suitable for investigating the antitumor activity of CRAd-synNotch and ADX730.

### T24 cells express HAS1, HAS3 and COX-2 mRNA

Some types of cancer cells express hyaluronan synthases (HAS1, HAS2, and HAS3), as well as fibroblasts and chondrocytes as part of the extracellular matrix, and the signaling between HA and CD44 is deemed critical for tumor proliferation and the spread of cancer. It has already been reported in previous studies that HAS1, HAS2, and HAS3 all synthesize HA [34, 35]. We compared the mRNA expressions of *HAS1*, *HAS2*, and *HAS3* in KK47, 5637, and T24 cells to those in SV-HUC-1 cells (non-malignant uroepithelial cells). As a result, *HAS1* and *HAS3* mRNA expression in T24 cells was significantly increased compared to SV-HUC-1 cells (*p* < 0.01; Fig. 2e). In 5637 cells, the mRNA expression of both HAS2 and HAS3 was significantly increased, whereas in KK47 cells, only the mRNA expression of HAS2 showed a significant increase (*p* < 0.01; Fig. 2e). Also, the expression of COX-2 in T24 cells was evaluated compared to BT474, a COX-2 negative breast cancer cell line. The mRNA expression of COX-2 in KK47, 5637, and T24 cells was significantly increased compared to BT474 cells, with a particularly high level observed in T24 cells (*p* < 0.01; Fig. 2d). These results indicated that KK47, 5637, and T24 endogenously expresses HA, and COX-2 is expressed in KK47, 5637, T24 but not in BT474.

### CRAd-synNotch specifically replicates in T24 cells

**Fig. 3:**
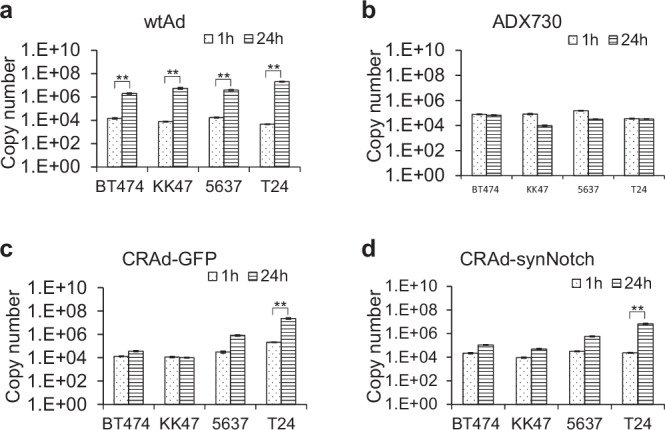
CRAd-synNotch replicates dependently on COX-2. The copy numbers of wild-type adenovirus (wtAd) (**a**), ADX730 (**b**), CRAd-GFP(**c**) and CRAd-synNotch (**d**) infected into BT474, KK47, 5637 and T24 cells were evaluated using absolute quantification by RT-PCR. All these results were expressed as the mean ± SE for three separate experiments (*p < 0.05, **p < 0.01). The standard curve was generated using the pAd1128 plasmid from the AdenoQuick 2.0 Kit (O.D.260Inc).

Four types of adenovirus, wtAd (wild type adenovirus type 5), ADX730, CRAd-GFP (recombinant adenovirus containing *E1* controlled by COX-2 promoter and *GFP* genes), and CRAd-synNotch were infected into KK47, 5637, T24 and BT474 cells, and their DNA copy numbers were evaluated by quantitative PCR. The copy numbers of wtAd significantly increased at 24 h infection compared to 1 h infection in COX-2 negative BT474 and COX-2 positive KK47, 5637, and T24 cells (*p* < 0.01; Fig. 3a), whereas the copy numbers of ADX730 did not increase in any of the cell lines (Fig. 3b). By contrast, the copy numbers of CRAd-synNotch and CRAd-GFP increased in T24 cells but not in KK47 and BT474 cells (*p* < 0.01; Fig. 3c, d). A replicating trend is observed in 5637 cells as well, but it was not statistically significant (*p* < 0.01; Fig. 3c, d). These results suggest that CRAd-synNotch, which contains the *E1* gene driven by COX-2 promoter, replicates in a COX-2-dependent manner. Therefore, we selected T24 cells and evaluated the antitumor effect of CRAd-synNotch in a mouse model.

### Both CRAd-synNotch and ADX730 transduce synNotch receptor protein in T24 cells

**Fig. 4:**
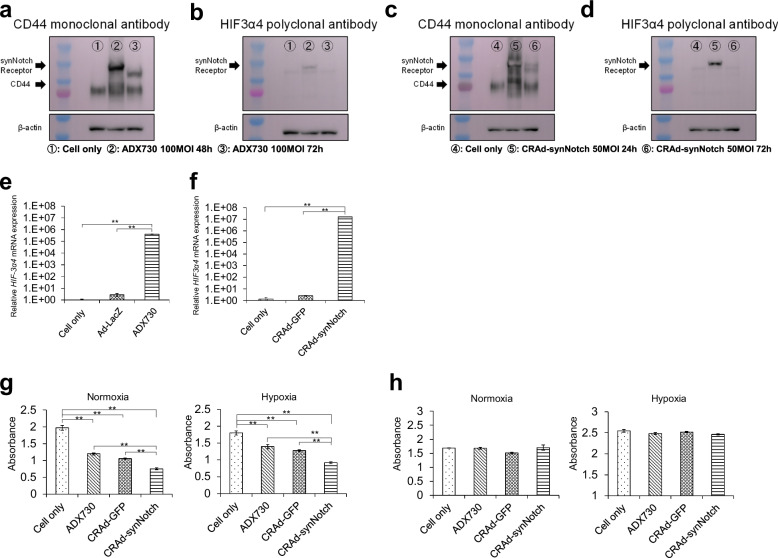
Both CRAd-synNotch and ADX730 transduce synNotch receptor fusion proteins in T24 cells and inhibits cell growth in T24 cells. The T24 cells were infected with ADX730 (**a**) at 100 MOI and CRAd-synNotch (**c**) at 50 MOI, and cultured for 24, 48, and 72 h. The expression of the synNotch receptor was analyzed by Western blotting using an anti-CD44 antibody. The T24 cells were infected with ADX730 (**b**) at 100 MOI and CRAd-synNotch (**d**) at 50 MOI, and cultured for 24, 48, and 72 h. The expression of the synNotch receptor was analyzed by Western blotting using a HIF-3α4 antibody. **e** The gene expression of HIF-3α4 in T24 cells was measured by real-time RT-PCR after Ad-lacZ and ADX730 infections. **f** The gene expression of HIF-3α4 in T24 cells was measured by real-time RT-PCR after CRAd-GFP and CRAd-synNotch infections. T24 (**g**) and BT474 (**h**) cells were treated with 50 MOIs of ADX730, CRAd-GFP, and CRAd-synNotch and incubated at 37°C for 72 h under oxygen concentrations of 2% (Hypoxia) or 21% (Normoxia). The absorbance was measured at a wavelength of 492 nm. All mRNA levels were standardized by the expression levels of the control gene TATA-binding protein (TBP) and analyzed using the ΔΔCt method. All these results were expressed as the mean ± SE for three separate experiments (**p < 0.01).

To confirm that CRAd-synNotch and ADX730 could transduce the synNotch protein, we performed a western blotting assay using anti-CD44 antibody and HIF3α4 antibody in 100 MOIs of ADX730 and 50 MOIs of CRAd-synNotch. As a result, strong expressions of endogenous CD44 proteins were observed in T24 cells. In addition to endogenous CD44 protein, the anti-CD44 monoclonal antibody detected CD44-ECD synthetic protein as a part of the synNotch protein in T24 cells infected with ADX730 for 48 h and 72 h, as well as with CRAd-synNotch for 24 h and 72 h (Fig. 4a, c). Interestingly, the synNotch proteins detected by the anti-CD44 antibody at 72 h after infection with CRAd-synNotch and ADX730 appeared to have lighter bands compared to the synthesized proteins at 24 h infection with CRAd-synNotch and 48 h infection with ADX730, respectively (Fig. 4a, c). These findings support the function of the synNotch protein, which releases HIF3α4 protein after binding with HA ligands. Using anti-HIF3α4 polyclonal antibody, the synNotch proteins were detected at 24 h after infection with CRAd-synNotch nor at 48 h after infection with ADX730; however, the synNotch protein was not detected at 72 h after infection with CRAd-synNotch and ADX730 (Fig. 4b, d). These findings were consistent with the results using anti-CD44 antibody. The anti-HIF3α4 antibody could detect HIF3α4 protein in the synNotch protein, but not the HIF3α4 protein after release from the synNotch protein.

### Both CRAd-synNotch and ADX730 induced *HIF-3α4* gene transduction

T24 cells were infected with the recombinant adenoviral vectors Ad-LacZ, ADX730, CRAd-GFP and CRAd-synNotch in vitro, and real-time RT-PCR was conducted to assess whether *HIF-3α4* genes were efficiently transduced or not. As a result, the mRNA expression of the *HIF-3a4* gene was significantly increased by infection with ADX730 (*p* < 0.01; Fig. 4e) and CRAd-synNotch (*p* < 0.01; Fig. 4f), compared to cell only (no treatment) and control adenovirus vector groups Ad-LacZ or CRAd-GFP.

### Both CRAd-synNotch and ADX730 inhibit cell growth of T24 cells but not that of BT474 cells

In the cell proliferation analysis, both CRAd-synNotch and ADX730 significantly inhibited the cell growth of T24 cells in normoxia and hypoxia conditions, compared to cell only (no treatment) groups. CRAd-synNotch exhibited a significantly higher anti-tumor effect than ADX730 and CRAd-GFP (*p* < 0.01; Fig. 4g). CRAd-synNotch and ADX730 did not show anti-tumor effects against BT474 cells (*p* < 0.01; Fig. 4h).

### Both CRAd-synNotch and ADX730 significantly suppressed CD44-downstream genes under hypoxia culture condition

**Fig. 5:**
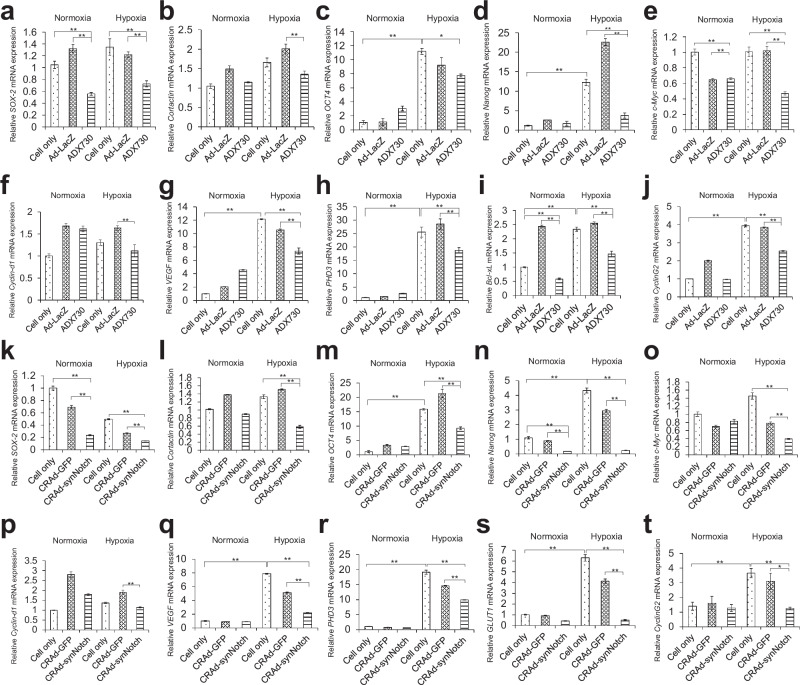
Gene expression of *SOX-2*, *Cortactin*, *OCT4*, *Nanog*, *c-Myc*, *Cyclin-d1*, *VEGF*, *PHD3*, *Bcl-xL* and *CyclinG2* in T24 cells infected with ADX730 and CRAd-synNotch. The mRNA expression of *SOX-2* (**a**) *Cortactin* (**b**) *OCT4* (**c**) *Nanog* (**d**) *c-Myc* (**e**) *Cyclin-d1* (**f**) *VEGF* (**g**) *PHD3* (**h**) *Bcl-xL* (**i**) and *CyclinG2* (**j**) levels in T24 cells infected with Ad-LacZ and ADX730 at 100 MOI were measured by real-time RT-PCR. The mRNA expression of *SOX-2* (**k**) *Cortactin* (**l**) *OCT4* (**m**) *Nanog* (**n**) *c-Myc* (**o**) *Cyclin-d1* (**p**) *VEGF* (**q**) *PHD3* (**r**) *GLUT1* (**s**) and *CyclinG2* (**t**) levels in T24 cells infected with CRAd-GFP and CRAd-synNotch at 50 MOI were measured by real-time RT-PCR. After viral infection, Cells were cultured under under oxygen concentrations of 2% or 21%. All mRNA levels were standardized by the expression levels of the control gene TATA-binding protein (TBP) and analyzed using the ΔΔCt method. All these results were expressed as the mean ± SE for three separate experiments (*p < 0.05, **p < 0.01).

T24 cells were infected with the Ad-LacZ, ADX730, CRAd-GFP, and CRAd-synNotch in vitro to examine by real-time RT-PCR whether ADX730 and CRAd-synNotch could suppress the downstream genes of CD44 via the CD44 decoy receptor function of the synNotch receptor. The relative mRNA expressions of *sex-determining region Y-box2 (SOX-2)*, *Cortactin*, *Octamer binding factor4* (*OCT4)*, *Nanog*, *Cellular Myelocytomatosis oncogene* (*c-Myc*) and *G1/S-specific cyclin-D1* (*Cyclin-d1*) downstream genes of CD44, in the cells infected with both ADX730 (*p* < 0.05 *p* < 0.01; Fig. 5a–f) and CRAd-synNotch (*p* < 0.01; Fig. 5k–p) were significantly lower than in cells infected with control adenovirus vectors (Ad-LacZ or CRAd-GFP) and cell only groups under culture conditions of hypoxia. The relative mRNA expressions of *SOX-2* levels infected with both ADX730 and CRAd-synNotch decreased even under normoxia conditions (*p* < 0.01; Fig. 5a and k) and the mRNA expressions of c-Myc levels infected with ADX730 also decreased even under normoxia conditions (*p* < 0.01; Fig. 5e). The mRNA expressions of *Nanog* levels infected with CRAd-synNotch also decreased even under normoxia conditions (*p* < 0.01; Fig. 5n), but other gene levels remained unchanged.

### The mRNA expression of HIF target genes in T24 was increased by hypoxia and significantly suppressed by both ADX730 and CRAd-synNotch

T24 cells were infected with the Ad-LacZ, ADX730, CRAd-GFP, and CRAd-synNotch in vitro to examine by real-time RT-PCR whether both ADX730 and CRAd-synNotch could suppress hypoxia target genes via the function of HIF-3α4 released from the synNotch receptor of CD44-N-HIF3α4 fusion protein. The mRNA levels of *vascular endothelial growth factor (VEGF)*, *Prolyl hydroxylase 3 (PHD3)*, *B-cell lymphoma-extra large (Bcl-xL)*, *CyclinG2 (CCND2)* and *Glucose transporter 1* (*GLUT1)* were significantly increased in 2% hypoxia culture compared to normoxia (*p* < 0.01; Fig. 5g–j and Fig. 5q–t). Under culture conditions of hypoxia, ADX730 significantly decreased the mRNA expression of *VEGF* (*p* < 0.01; Fig. 5g), *PHD3* (*p* < 0.01; Fig. 5h), *Bcl-xL* (*p* < 0.01; Fig. 5i), and *CyclinG2* (*p* < 0.01; Fig. 5j) compared to the Ad-LacZ and cell only groups. The relative mRNA expressions of *Bcl-xL* levels decreased even under normoxia conditions, but other gene levels remained unchanged (p < 0.01; Fig. 5i). CRAd-synNotch significantly decreased the mRNA expression of *VEGF* (*p* < 0.01; Fig. 5q), *PHD3* (*p* < 0.01; Fig. 5r), *GLTU1* (*p* < 0.01; Fig. 5s), and *CyclinG2* (*p* < 0.01; Fig. 5t) compared to the CRAd-GFP and cell only groups under culture conditions of hypoxia.

### Intratumoral injections of CRAd-synNotch and ADX730 induced CD44 overexpression in the cell membrane in T24 xenograft tumors in vivo

**Fig. 6:**
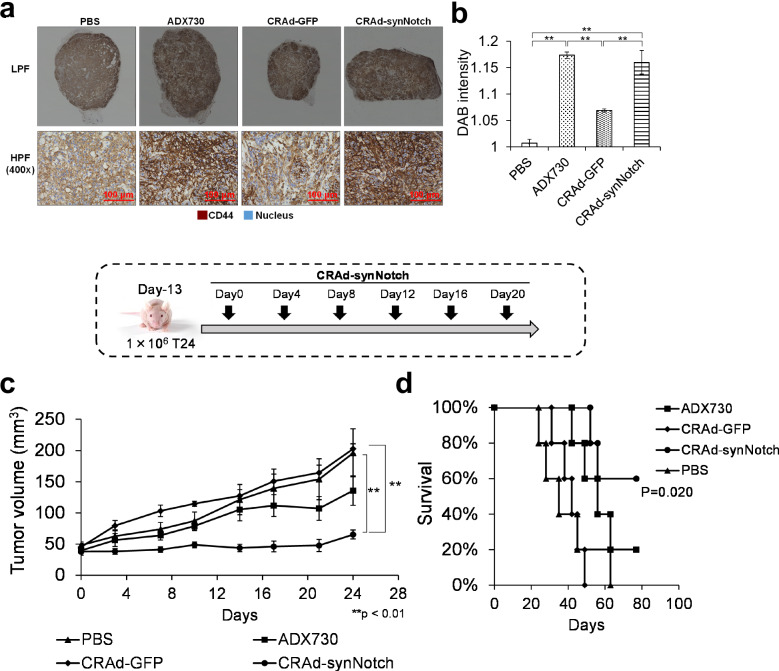
Immunohistochemical staining for CD44 and antitumor effect of CRAd-synNotch in mice with T24 tumors. **a** Immunohistochemical staining image after subcutaneously injecting T24 cells into nude mice and infecting them with PBS, ADX730, CRAd-GFP, and CRAd-synNotch; HPF: High Power Field, Original magnification: x400; LPF: Low Power Field, Original magnification: x80 (*n* = 3 per group, average ±SE bars, **p < 0.01). **b** Quantitative analysis of (**a**) using Visiopharm Oncotopix Discovery software. DAB: 3,3’-diaminobenzidine. **c** Tumor growth curve after subcutaneously injecting T24 cells into nude mice and infecting them with PBS, ADX730, CRAd-GFP, and CRAd-synNotch (*n* = 5 per group, average ±SE bars, **p < 0.01). **d** Survival in the T24 nude mouse model (*n* = 5 per group).

Immunohistochemical analysis revealed positive CD44 protein expression on the cell membranes of T24 tumors across all treatment groups. Notably, the most intense staining occurred in tumors treated with CRAd-synNotch and ADX730 (Fig. 6a, b). These findings align with the increased CD44 expression confirmed by Western blot analysis (Fig. 4a, c), demonstrating that both CRAd-synNotch and ADX730 treatments are capable of inducing elevated levels of CD44-ECD as a part of synNotch protein on the surface of tumor cells.

### Intratumoral injections of CRAd-synNotch significantly suppressed the growth of T24 xenograft tumors in mice and prolonged their survival

The recombinant adenovirus vectors, ADX730, CRAd-GFP, and CRAd-synNotch, and PBS were intratumorally injected into the T24 subcutaneous xenograft tumors in mice to examine the in vivo anti-tumor activity of CRAd-synNotch in comparison with CRAd-GFP, ADX730 and PBS groups. Briefly, the intratumoral injection of each adenoviral vector was initiated on Day 13 after the T24 cell inoculation. Subsequently, the adenovirus vectors or PBS were injected once every 3 days, for a total of 6 times. At the day 24 after the start day of injections, CRAd-synNotch significantly suppressed tumor growth compared to the CRAd-GFP and PBS groups (*p* < 0.01; Fig. 6c). No significant difference was observed between the CRAd-syNotch and the ADX730 groups; however, CRAd-synNotch exhibited higher anti-tumor activity compared to ADX730. Furthermore, continued observation of survival revealed a significant difference in the group treated with CRAd-synNotch compared to the CRAd-GFP, ADX730, and PBS groups (*p* < 0.05; Fig. 6d).

## Discussion

In this study, we composed a conditionally replicative adenovirus vector (CRAd-synNotch) that carries the adenoviral E1 gene controlled by COX-2 promoter, the *synNotch receptor* gene, and the *Ad5/35 fiber* gene (Fig. 1c). Recently, the development of oncolytic adenoviruses and their implementation in clinical trials has gained increased attention. Gene therapy using replication-deficient adenovirus is limited to the expression of introduced therapeutic genes, while in suicide gene therapy it is limited to the destruction of infected cells and surround cells. In contrast, with the oncolytic approach, the virus replicates, followed by the lysis of infected cells, and new infectious units are released directly into the tumor environment. An important aspect of tumor collapse by replicating adenovirus is that it is effective against tumor stem cells, which often cause tumor recurrence due to intrinsic resistance to therapy [36]. Furthermore, oncolytic adenoviruses have the ability to provoke immunogenic cell death (ICD). When cells undergo lysis, offspring viruses, pathogen-associated molecular patterns (PAMPs), damage-associated molecular patterns (DAMPs), and tumor-associated antigens (TAAs) are dispersed into the tumor microenvironment (TME). This release of offspring viruses continues the infection cycle by targeting previously uninfected cancer cells, thus promoting the spread of the virus. The immunostimulatory molecules that are emitted into the TME draw immune cells, which then trigger both the innate and adaptive immune systems to combat the tumor [37].

In recent years, researchers have developed synNotch receptors to create artificial Notch signaling pathways [25, 38, 39]. Roybal and colleagues [36] previously engineered T cells equipped with synNotch receptors where the extracellular domain (ECD) of Notch was substituted with a single-chain variable fragment (scFv) targeting cancer antigens like CD19 and HER2, and the intracellular domain (ICD) was replaced with a fusion of the Gal4 DNA binding domain and the VP64 tetrameric viral transcription activator domain [40]. These synNotch receptors enabled the engineered T cells to attach to specific antigens on cancer cells, successively initiating gene transcription that activates T cells or triggers apoptosis in cancer cells [38]. In addition, we previously demonstrated that recombinant adenovirus vector ADX730 containing *synNotch receptor* (CD44-N-HIF3α4) gene could inhibit the growth of human triple-negative breast cancer cells [25]. The present study aimed to enhance the anti-tumor activity of ADX730 by using a conditionally-replicative adenovirus platform and investigate its antitumor effects.

To evaluate the antitumor activity of both CRAd-synNotch and ADX730 in MIBC, we employed a T24 MIBC cell line. T24 cells expressed CAR and CD46 (Fig. 2a), suggesting the presence of receptors for adenoviruses with Ad5/35 fiber on the surface of T24 cells. Therefore, we considered that CRAd-synNotch and ADX730 are capable of infecting T24 cells. T24 cells also express CD44, HAS1, and HAS3 (Fig. 2e, f). HA-CD44 signaling is considered to play a vital role in tumor growth and metastasis [34, 35]. Indeed, in our experiments both CRAd-synNotch and ADX730 could transduce synNotch protein expression in T24 cells (Fig. 4a, c), and through the synNotch system HIF3α4 can be released from the synNotch protein (Fig. 4b, d). The anti-HIF3α4 antibody could detect HIF3α4 protein in the synNotch protein, but not the HIF3α4 protein after release from the synNotch protein. HIF-3α4 competes with HIF-1α to inhibit hypoxia response signaling. It is believed that after fulfilling its role, HIF-3α4 is degraded through the ubiquitin-proteasome system, which may explain why it is not detectable. Although further research is needed to clarify the details of HIF-3α4’s role and its subsequent fate, it is generally understood that the expression and stability of HIF-3α4 are tightly regulated, and unnecessary HIF-3α4 is promptly degraded through specific mechanisms [17]. The reason the detection band of HIF-3α4 appears fainter compared to CD44 (Fig. 4a, c) is thought to be because some of the HIF-3α4 has already been degraded by the ubiquitin-proteasome system. The cell growth of T24 cells was inhibited by CRAd-synNotch and ADX730 (Fig. 4g). These results suggested that CRAd-synNotch and ADX730 specifically work in CD44 over-expressing cancer cells [25].

COX-2 is primarily responsible for prostaglandins produced in inflammatory sites, and is virtually undetectable in most tissues under physiological conditions [26]. In contrast, recent studies demonstrated that COX-2 is expressed in several cancer tissues and may have an important role in carcinogenesis, including breast, colon, and lung cancers, as well as bladder cancer [41]. The tumor-specific expression of COX-2 suggested to us the potential utility of COX-2 promoter for the construction of a novel replication-selective adenovirus to treat bladder cancer. To demonstrate that CRAd-synNotch proliferates dependently on COX-2, the relative increase in virus levels was quantified in KK47, 5637, T24, and BT474 cells. It is known that COX-2 protein expression is negative in BT474 cells. Quantitative PCR results showed a significant increase of CRAd-synNotch and CRAd-GFP at 24 h infection compared to immediately after infection in T24 cells, but not in BT474 and KK47 cells (Fig. 3c, d). CRAd-synNotch appears to replicate less than CRAd-GFP in T24 cells. The reason for this may be that both CRAd-synNotch and CRAd-GFP are inserted into the E3 region of the adenovirus, but the length of the synNotch gene is 2856 bp, whereas the length of the GFP gene is 720 bp. Therefore, it is considered that the gene length may affect the replication speed. A replicating trend is observed in 5637 cells as well, but it was not statistically significant (Fig. 3c, d). Conversely, wtAd at 24 h of infection compared to immediately after infection significantly increased in KK47, 5637, T24 and BT474 cells (Fig. 3a). wtAd has an E1 promoter upstream of the E1 region of the genome and can proliferate independently of COX-2. On the other hand, CRAd-synNotch and CRAd-GFP have a COX-2 promoter incorporated upstream of the E1 region and are activated only in the presence of COX-2 protein. Thus, CRAd-synNotch and CRAd-GFP infect T24 cells with high COX-2 expression, proliferate dependently on COX-2 within the cells, and initiate tumor oncolysis. Although it has been reported in previous studies that COX-2 is expressed in bone marrow cell lines, but its expression is nearly absent compared to multiple myeloma or cancer cells [42]. The data in Fig. 2f and Fig. 3 indicate that CRAd-synNotch replicates in a COX-2-dependent manner and suggest that it does not replicate in bone marrow cell lines with low COX-2 expression. To evaluate the efficacy of CRAd-synNotch and ADX730, we compared in vivo antitumor activities with control adenovirus vectors and PBS groups. It is well known that interaction between CD44 and HA enhances Ad-vector infectivity [43].

First, we confirmed that both CRAd-synNotch and ADX730 could efficiently transduce *HIF-3α4* gene in T24 cells (Fig. 4e and f). To confirm the function of CRAd-synNotch and ADX730, we examined the function of CRAd-synNotch and ADX730 under hypoxia conditions in vitro in T24 cells. We confirmed that CRAd-synNotch and ADX730 significantly suppressed the mRNA expressions of *SOX-2*, *Cortactin*, *OCT4*, *Nanog*, *c-Myc* and *Cyclin-d1*genes, which are CD44-downstream target genes, compared to the control adenovirus vectors and cell only (no treatment) groups (Fig. 5a–f and Fig. 5k–p). SOX-2, a transcription factor, is closely linked with the stem-like properties of cancer cells [44]. Multiple studies have shown that tumor-associated macrophages (TAMs) can directly influence the expression of cancer stem cells (CSCs) and their markers, specifically regulating SOX-2 [45]. Furthermore, cortactin is key in controlling the strength of intercellular adhesion through N-cadherin [46], and high cortactin levels have been clinically linked to a worse prognosis or recurrence in patients [47]. OCT4, in conjunction with (c)-MYC, Krüppel-like factor 4 (KLF4) and SOX2, plays a crucial role in the induction of pluripotency in both human and mouse somatic cells [48] and has been identified as a key factor in driving the properties of cancer stem cells (CSCs), with extensive research highlighting its importance for CSC self-renewal and pluripotency [49]. The presence of Nanog has been documented across a range of human cancers, with high expression levels often associated with more advanced disease stages, poor differentiation, and decreased overall survival rates [50]. Studies of renal cell carcinoma and urothelial carcinoma revealed that up to 91% of cancer cells in MIBC exhibited high levels of Nanog expression [51]. The proto-oncogene c-Myc encodes the c-Myc protein, which regulates numerous target genes and is crucial for processes like cell growth, proliferation, metabolism, apoptosis, and angiogenesis. Overexpression of Myc is reported in BC and is a well-known cause of carcinogenesis, making it one of the most commonly dysregulated oncogenes in human cancers [52]. Cyclin-d1 plays a crucial role in CSCs by promoting cell cycle progression and supporting self-renewal and proliferation. In CSCs, it aids the G1 to S phase transition, essential for growth and survival. Cyclin-d1 also participates in pathways that maintain CSC stemness, contributing to therapy resistance and tumor relapse. Elevated Cyclin-d1 expression in CSCs is linked to aggressive tumor behavior and poor prognosis [53].

Also, we examined the effects of CRAd-synNotch and ADX730 on HIF target genes, VEGF, PHD3, Bcl-xL, CyclinG2, and GLUT1. VEGF plays a crucial role in tumor angiogenesis and is identified as a specific target for HIF-1α [54]. There is a notable correlation between VEGF expression and tumor grade, along with a negative correlation with the depth of muscle invasion [55]. PHD3 stands out among the critical molecules that are upregulated in hypoxic conditions and is highly expressed in pancreatic ductal adenocarcinoma, affecting patient survival. Studies have shown that patients with PHD3-positive papillary breast carcinoma have significantly poorer survival rates compared to those with PHD3-negative papillary breast carcinoma [56]. Our data showing high expression of PHD3 under hypoxic conditions suggest the potential involvement of PHD3 in the proliferation of bladder cancer (Fig. 5h and r). Typically, cytotoxic drugs are known to trigger apoptosis in cancer cells; however, in particular cancer cell types, cytotoxic agents can also lead to the upregulation of anti-apoptotic proteins such as Bcl-xL [57]. The chemoresistance driven by HIF may be facilitated by such anti-apoptotic proteins, including Bcl-xL [58]. It is widely accepted that overexpression of CyclinG2 tends to enhance oncogenic activities across various cancers [59]. Furthermore, the silencing of CyclinG2, through methylation of its promoter region, is linked to the advancement of numerous cancers, including bladder cancer [60]. While normal bladder mucosa does not show GLUT1 expression, it is evident in malignant bladder tissues, with higher levels seen in muscle-invasive cancers than in non-muscle-invasive ones [61]. The expression of GLUT1 is considered an inherent characteristic of hypoxia in cancers, including bladder cancer [62], and its co-expression with pimonidazole (a marker of external hypoxia) has been observed [63]. In our results, the expressions of VEGF, PHD3, Bcl-xL, and CyclinG2 genes were significantly suppressed by ADX730 compared to the control adenovirus vectors under culture condition of hypoxia (Fig. 5g–j). The expression of VEGF, PHD3, GLUT1 and CyclinG2 genes were significantly suppressed by CRAd-synNotch compared to the control adenovirus vectors under culture conditions of hypoxia (Fig. 5q–t).

In an in vivo study, we employed a mouse xenograft model of T24 tumor. The over-expression of CD44-ECD induced by CRAd-synNotch and ADX730 was clearly observed in immunohistochemical studies (Fig. 6a, b), and CRAd-synNotch exhibited greater anti-tumor efficacy compared to PBS and CRAd-GFP groups in the T24 mouse model (Fig. 6c, d). In this study, the xenograft model employing immunodeficient nude mice presented certain limitations. Specifically, since multiple injections of Ad-vectors were administered, we were unable to assess the immune responses to both the vectors and the transgenes. Consequently, it remains crucial to determine whether these immune responses to the Ad-vectors in human clinical trials could amplify or diminish the observed anti-tumor effects. In this study, we were also unable to clearly demonstrate the action of the Ad5/35 fiber. However, when comparing our experimental results in Fig. 4b, d with Fig. 4e, f, it is clear that the gene transduction efficiency has significantly increased. Moreover, as the purpose of this study is to show that CRAd-synNotch replicates in a COX-2-dependent manner and exhibits antitumor effects against MIBC, we have left the role of the Ad5/35 fiber as a subject for future research.

In 2022, Adstiladrin (nadofaragene firadenovec), a viral therapy for NMIBC that carries human IFNα2b cDNA in an adenoviral vector, was approved in the United States. The mechanism of action of this therapy involves injecting the adenoviral vector into the bladder, where IFNα2b cDNA is introduced into bladder epithelial cells via CAR. This is then transcribed into the IFNα2b protein, which exerts direct and indirect immunomodulatory effects to inhibit tumor growth and treat cancer. Like Adstiladrin, this study also uses an adenoviral vector, but it targets cancer stem cells and has been modified with a type 35 adenovirus fiber that binds to CD46, which is expressed by most tumor cells as well as CAR. This allows the treatment to be effective even on tumor cells that do not express CAR, thereby enhancing the efficiency of restricted proliferation. While Adstiladrin is intended for NMIBC, this study targets the more challenging MIBC.

In conclusion, we developed a conditionally replicative adenovirus vector, CRAd-synNotch, and a recombinant adenovirus vector, ADX730, containing a synNotch receptor gene that inhibits CD44 signaling and hypoxia-induced response in cancer cells. Both CRAd-synNotch and ADX730 work under conditions of normoxia and hypoxia but are especially effective under conditions of hypoxia and the presence of HA in vitro, and greatly inhibited the growth of T24 MIBC tumors in vivo. CRAd-synNotch and ADX730 are completely novel gene therapy drugs targeting cancer stem cells, and show high clinical applicability for MIBC, especially CRAd-synNotch.

## Data Availability

The datasets generated and/or analyzed during the current study are available from the corresponding author on reasonable request.
